# Justifying the unethical: Are we following the wrong trend regarding authorship? Perspective from Pakistan

**DOI:** 10.1016/j.amsu.2022.103946

**Published:** 2022-06-06

**Authors:** Khawar Abbas, Sajeel Saeed, Kashif Tousif, Mohammad Ebad ur Rehman

**Affiliations:** Department of Surgery, Rawalpindi Medical University, Block E Satellite Town, Rawalpindi, Pakistan; Department of Medicine, Rawalpindi Medical University, Block E Satellite Town, Rawalpindi, Pakistan

**Keywords:** Authorship, Unethical practice, Researcher, Research

Dear Editor,

Since interdisciplinary collaboration has become more widespread in practically every scientific field and the race for publication glory, the dispute over the definition, eligibility for, and order of authorship is never-ending. Authorship credit has significant intellectual, social, and commercial ramifications. The contribution of each author who has made important scientific contributions to a work should be used to determine authorship. Throughout the study process, transparency on authorship issues is required, and any dispute should be handled openly with all project participants. Authorship determination is a dynamic procedure that should never be based on a preset choice and includes all participants in the research.

The prevailing culture of ‘publish or perish’ compels authors to increase their quantity of work without focusing on the quality and secondly many medical journal editing agencies determine authorship qualifications depending on the quantity of research work. However, occasional discredits have occurred and pressure from a senior member leads to an undue alteration of the author positions. Also, students unwillingly make supervisors leading authors due to their authority and this thing justifies the notion of coercion/hostage authorship. It can be corrected when junior researchers will excel in terms of experience, knowledge, and morale [1].

Some students give honorary or gift authorship to their supervisors as first author or coauthor. Maybe the supervisor didn't do a significant contribution to the research project but junior researchers do this out of benefit associated with it as this increases the chances of getting published and makes their work more credible. Despite offering leading authorship to supervisors, students themselves need to take the forefront in their research works and nurture themselves to abstain from this type of practice [2].

In developing countries like in our setting where there is less advancement in the research field as compared to the Western world, regional authors want to collaborate or they give preference to co-author with high-ranked authors of Western research institutions which automatically promotes honorary authorship. Also, there is a high prevalence of non-compliance to authorship guidelines in non-indexed journals [3].

There is also an unethical practice common among the researchers that are to place each others’ names in their respective researches without taking any part during the research procedure which is known as mutual support authorship. The purpose is clear that is to increase the number of publications. Research scrutinizing bodies can correct this wrong exercise by implementing effective policies for determining the real value of research by an author [4].

All these unethical authorship practices give rise to multi-authored research. Multi-authorship somewhat can be justified because of the integrated and multi-disciplinary approach in medical research but most of the time the reason is simple which had been discussed previously and this is pressure to publish. So, the major concern with multi-authorship is that the honorary or mutual support authors will gain benefits from obtaining grants to becoming reviewers or editors and the vicious cycle will start which ultimately harms the research integrity and scientific appropriateness [5].

To overcome this conundrum, the International Committee of Medical Journal Editors (ICMJE) Elsevier, Credit Taxonomy, McNutt authorship guide, and even the Pakistani Higher Education Commission (HEC) have created various authorship standards [[6], [7], [8], [9]]. Despite these guidelines, violations of authorship remain prevalent, and many researchers violate these principles in a sizable proportion of peer-reviewed medical journals.

These all rules suggest only one thing be certain: the one who conceptualizes and does the most amount of work should be regarded as the first author and so on. Yet the problem remains the same, as many early career researchers face: what would be the proof who did the most amount of work in the project, is this important to add supervisors, members from the department from where we are collecting the data, cross crediting with other researchers for the sake of increment of publications or mentioning someone else's name in our research and regard him/her as the first author without his/her contribution in that project to get future promotions or benefits?

Authorship's unethical behaviours cannot be resolved just by the establishment of rules. The current solicitation for publications that will be utilised to determine an individual's rank/promotion must be addressed. The academic system should modify itself from meriting researchers based on their publications. Research societies can play a constructive role in this regard by promoting research and publication ethics among young researchers. Educational institutions and seniors can educate and train their students and incorporate research ethics in their curricula to follow the authorship ethos [10].

## Ethical approval

Not applicable.

## Funding

No funding is required for the study.

## Consent

Not required.

## Registration of research studies

1. Name of the registry: Not applicable
2. Unique Identifying number or registration ID: Not applicable
3. Hyperlink to your specific registration (must be publicly accessible and will be checked): Not applicable

## Provenance and peer review

Not commissioned, externally peer reviewed.

## Author contribution

Khawar Abbas: Study conception, write-up, critical review and approval of the final version, Sajeel Saeed: Study conception, write-up, critical review and approval of the final version, Kashif Tousif: Study conception, write-up and approval of the final version, Mohammad Ebad ur Rehman: Study conception, critical review and approval of the final version.

## Guarantor

Khawar Abbas: Department of Surgery, Rawalpindi Medical University, Block E Satellite Town, Rawalpindi, Pakistan.

Sajeel Saeed: Department of Medicine, Rawalpindi Medical University, Block E Satellite Town, Rawalpindi, Pakistan.

## Declaration of competing interest

All authors declared no conflict of interest.
